# A Pilot Study to Explore Patient Satisfaction With a Virtual Rehabilitation Program in Multiple Sclerosis: The RehabVR Study Protocol

**DOI:** 10.3389/fneur.2020.00900

**Published:** 2020-08-21

**Authors:** Virginia Meca-Lallana, Daniel Prefasi, Walter Alabarcez, Teresa Hernández, Fabiola García-Vaz, Angélica Portaña, David Gomis, Nieves Téllez, Cristina García-Bernáldez, Jorge Mauriño, Nicolás Medrano, Aránzazu Vázquez-Doce

**Affiliations:** ^1^Neurology Department, Hospital Universitario de La Princesa, Madrid, Spain; ^2^Medical Department, Roche Farma, Madrid, Spain; ^3^La Frontera, Madrid, Spain; ^4^Rehabilitation Department and Physiotherapy Unit, Hospital Universitario de La Princesa, Madrid, Spain; ^5^Biomedical Research Foundation, Hospital Universitario de La Princesa, Madrid, Spain; ^6^Hospital Clínico Universitario, Valladolid, Spain; ^7^Physical Medicine and Rehabilitation Department, Hospital Universitario de La Princesa, Madrid, Spain

**Keywords:** rehabilitation, virtual reality, multiple sclerosis, patient satisfaction, adherence

## Abstract

**Background:** Virtual reality (VR) has emerged as a promising treatment approach in rehabilitation for patients with multiple sclerosis (MS) due to its potential to increase patient motivation and rehabilitation adherence. One of the key features for rehabilitation adherence is patient satisfaction with the VR rehabilitation (VRR) program, and information on user satisfaction and not only effectiveness is required to systematically include VRR in routine clinical practice. Given that information on patient satisfaction with VRR is scarce, the primary objective of this study is to assess long-term patient satisfaction with a novel VRR program. This program has been specifically designed for MS patients by a multidisciplinary team of specialists, based on an effective conventional rehabilitation (CR) program. Secondarily, discomfort with VRR will be evaluated, and therapy adherence and changes in a variety of domains typically affected by MS will be compared between patients receiving VRR and patients receiving CR.

**Methods:** In this prospective single-center 6-months follow-up study, 32 and 16 MS patients will receive VRR or CR, respectively. Patients will attend twice weekly rehabilitation sessions on site during 4 weeks, and they will continue with rehabilitation at home for five additional months. Satisfaction, assessed by the User Satisfaction Evaluation Questionnaire (USEQ), at 6 months of the VRR program initiation will be the primary outcome. Secondary outcomes include adherence, disability, spasms and spasticity, balance, fatigue, activities of daily living (ADLs), depression, anxiety, work status, cognition, demographic, and clinical characteristics (in the VRR and CR groups), and discomfort (in the VRR group). Outcome measures will be assessed at baseline, and at 1 and 6 months of rehabilitation initiation.

**Discussion:** The study is intended to provide a better understanding of long-term patient satisfaction with a VRR program specifically designed for MS patients. Additionally, the study will provide information on long-term adherence, changes in motor symptoms, cognitive functions and patient-reported outcomes after the rehabilitation program. The results from this study will help to gather valuable knowledge on the use of rehabilitation with a new VR tool in MS patients.

## Introduction

Multiple sclerosis (MS) is the most frequent cause of non-traumatic neurological disability in young adults in developed countries. Its prevalence has substantially increased in the last three decades and it affects ~2.3 million people worldwide (1, 2). The disease is associated with a high economic burden for society, and Spain is the European country with the highest total annual cost per patient (3, 4).

The clinical presentation of MS is heterogeneous and depends on the location of demyelinating lesions within the central nervous system. The most frequent symptoms and signs observed at disease onset originate from the optic nerve, the brainstem or cerebellum, the spinal cord, and the cerebral hemispheres (5). Motor manifestations (such as spasticity, gait, and balance impairments), optic neuritis, and sensory symptoms appear early in the disease course (6), and usually lead to progressive limitation of daily life activities. The majority of patients at the initial phase of MS have reversible episodes of neurological deficits (known as relapses) that usually last for days or weeks (relapsing-remitting MS; RRMS). Over time, in a proportion of patients, the development of permanent neurological deficits and the progression of clinical disability become prominent (secondary progressive MS; SPMS). A minority of patients have a progressive disease course from onset (primary progressive MS; PPMS). Most patients with MS, regardless of the initial disease course, will eventually require assistance to walk (7). It has been estimated that the average disease duration from diagnosis to use of a permanent walking aid is ~13 years (8). In fact, these motor problems have recently been identified by MS patients as the symptoms most undermining their health-related quality of life (HRQoL) (9).

Along with motor limitations, up to 65% of MS patients present impairments in a variety of cognitive domains within a year of diagnosis, processing speed being the most frequently affected domain in this time period (10). Deficits in memory, attention, executive functions, and visual perceptual functions are also prevalent in a considerable number of patients (11). The combination of all these impairments poses a threat to patients' daily life and is associated with work difficulties and negative outcomes (12, 13), including an early loss of productivity (14).

Despite continuous advances in MS management and the availability of increasingly effective disease-modifying therapies (DMTs), patients still have a high disability burden over long periods of time. This increased period of disease disability provides at the same time greater potential for rehabilitative therapies to reduce impairment through the strengthening of residual capacities and learning of new strategies. Rehabilitation consists of individualized and goal-oriented tasks aimed at improving functional independence. A review of reviews on rehabilitation in MS found strong evidence for the benefits of physical therapy on function and participation (15). Cognitive rehabilitation, on the other hand, has also shown improvements in cognitive abilities (16), although the evidence is not as conclusive as for motor rehabilitation.

In general, motor and cognitive rehabilitation therapy poses some significant challenges. On one hand, attending rehabilitation sessions on a regular basis might be difficult due to MS patient mobility difficulties, geographical location, and/or limited resources. On the other hand, traditional rehabilitation exercises are usually repetitive and tedious, which might decrease the patient's interest and could lead to reduced exercise adherence. Virtual reality rehabilitation (VRR) has been proposed as a promising therapeutic tool to overcome the drawbacks of conventional rehabilitation (CR).

Virtual reality rehabilitation offers the possibility of task-oriented and home-based training that allows the simulation of complex multisensory situations simulating everyday activities. The use of VR with lifelike scenarios in people with MS has shown to be safe and effective in motor rehabilitation, including gait, balance and arm mobility improvements (17–23). VRR not only engages patients in motor activities, but it simultaneously require patients to use cognitive abilities, since enriched virtual environments usually require greater attention and dual-tasking to complete the goal-oriented task (22).

Due to the stimulating and interactive nature of virtual environments in which rehabilitation exercises are presented as games, VRR has the potential to be a useful tool to increase motivation and therapy adherence (24). One of the key features for rehabilitation adherence is patient satisfaction with the program. In line with this, a study has recently shown that MS patient satisfaction with a telerehabilitation program was the only factor that significantly differentiated between low and high adherence groups, while other factors such as fatigue, disability, physical and psychological impact of the disease, sleep quality, and HRQoL, among others, were not different between the two groups (25). The study also demonstrated that patient satisfaction with the program was a predictive factor for high rehabilitation adherence (25).

Despite VRR programs having been shown to be feasible and accepted by MS patients (17, 19, 23, 24), the evidence on MS patient satisfaction with VRR programs is scarce (26). Satisfaction, together with effectiveness and efficiency in a quantified context of use, define the usability of a system or product (27). Several questionnaires have been designed to evaluate usability in computer systems (28), but as far as we know, only one questionnaire so far has been validated to address satisfaction of use in VRR programs: the User Satisfaction Evaluation Questionnaire (USEQ) (29).

Importantly, most of the studies evaluating VRR programs in MS have used technologies such as Nintendo Wii Balance Board System and Microsoft Kinect Sensor Xbox 360 (22, 24, 30–33), which were created for entertainment of the general population (exergames) and were not specifically designed for rehabilitation of impaired functions (serious games). Furthermore, the feasibility of VRR in MS has usually been assessed after a 6-weeks (18, 19, 26, 34) or 10-weeks program (24), which might not be enough time to confirm patient acceptance of VRR in the long term.

There is presently limited evidence on MS patient satisfaction with VRR programs using gamified exercises to target MS needs. A better understanding of long-term patient satisfaction with a VRR program specifically designed by a multidisciplinary panel of experts in MS and experts in gamification, immersive narrative and interactive engineering, would allow customization of the program to patient needs, which would likely increase rehabilitation adherence, and hence improve clinical outcomes.

The primary aim of this pilot study is to evaluate satisfaction with a VRR program in patients with MS at 6 months of program initiation.

Secondarily, we aim to: (i) describe adherence to a VRR and a CR program during 6 months; (ii) compare changes after 6 months of VRR or CR in a variety of domains typically affected by MS, including HRQoL, fatigue, activities of daily living (ADLs), work status, depression, anxiety, disability, spasms and spasticity, balance, and cognition; (iii) evaluate the presence of discomfort during the VRR program; (iv) explore clinical and demographic characteristics associated with patient satisfaction with the VRR program.

An exploratory data analysis of the secondary objectives described above will be performed at 1 month of rehabilitation program initiation.

## Methods

### Study Design

**Figure 1:**
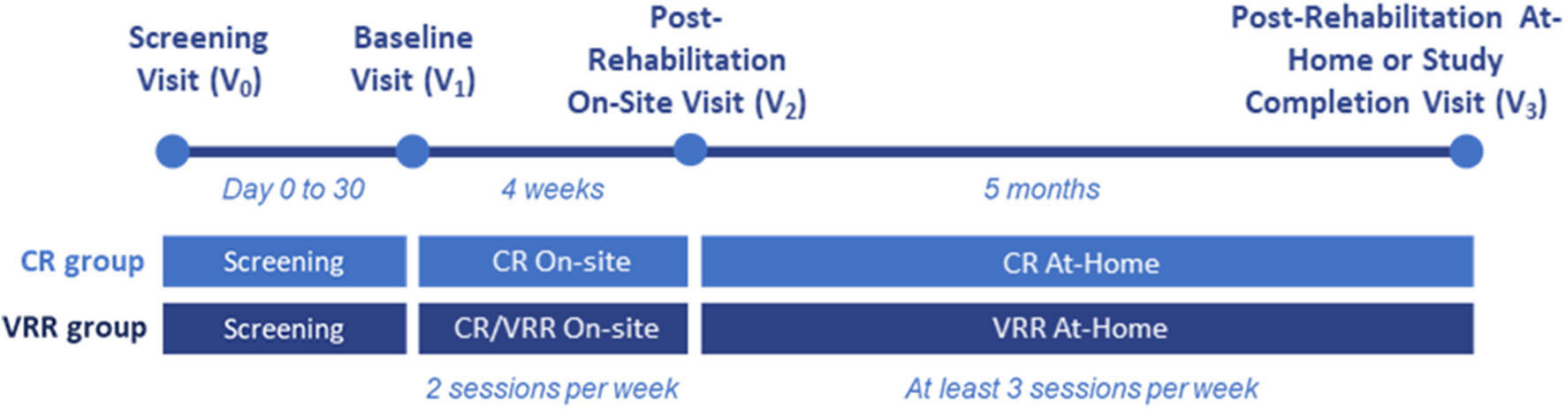
Study design.

To test the study objectives, a prospective single-center 6-months follow-up study will be conducted in patients with MS. During this period, MS patients will be treated and/or evaluated by a multidisciplinary team of specialists including neurologists, physiotherapists, physiatrists, neuropsychologists and nurses. After patients have signed the informed consent form and have been confirmed to meet the selection criteria, they will be randomized 2:1 to the VRR group or the CR group. This unequal ratio has been used so that each subject entering the study has twice the probability of being assigned to the VRR group, which is expected to positively impact the patient experience with the rehabilitation program. In the VRR group, patients will receive 4 CR sessions plus 4 VRR sessions at the hospital during ~4 weeks. In the CR group, patients will receive 8 sessions of CR at the hospital during ~4 weeks. After the on-site rehabilitation, both groups will continue with rehabilitation at home for an additional 5 months. The VRR group will receive a VR headset and the CR group will receive instructions on CR techniques and a notebook with cognitive tasks. Figure 1 shows an overview of the study design.

The randomization lists will be created using the EPIDAT statistical software developed by the *Servicio de Información sobre Saúde Pública de la Conseller*í*a de Sanidade e Servicios Sociais de la Xunta de Galicia* (Spain) and the *Special Program for Health Analysis* (SHA) of the Pan American Health Organization (PAHO). A permuted block design with a computer random number generator will be used. The neurologist, physiatrist, and neuropsychologist involved in the assessment of the patient will be blinded to randomization. The physiatrist, physiotherapist, nurse, and neurologist treating the patient will be aware of the randomization. Both randomization and blinding are not required to achieve the primary aim of the study. However, we decided to randomize the patients to prevent selection bias and allow comparisons between groups (secondary aims). Blinding was also considered to be appropriate in order to increase the objectivity of the neurologist and neuropsychologists involved in patient assessment.

The number of study visits will be up to 4. A screening visit (V_0_) where the patient will be invited to participate in the study after being informed of its characteristics, a baseline visit (V_1_) where training on the VRR or CR will be conducted, a post-rehabilitation on-site visit (V_2_), and a post-rehabilitation at-home visit or study competition visit (V_3_). All visits will be conducted at the time of routine clinical practice visits. The screening visit (V_0_) and baseline visit (V_1_) might be combined, and in that case the total number of study visits will be 3.

The study was approved by the Ethics Committee of the Hospital Universitario de la Princesa on 26/09/2019 and will be conducted in accordance with the ethical standards laid down in the 1964 Declaration of Helsinki.

### Participants

The multidisciplinary team of specialists conducting the study will invite all consecutive MS patients attending their regular clinical follow-up visits at the Hospital Universitario de la Princesa in Madrid (Spain) to participate in the study. The participants will be screened for eligibility by the study supervisor according to the inclusion and exclusion criteria. Recruitment started in January 2020.

### Eligibility Criteria

Participants are eligible for the study if they meet the following inclusion criteria:

1. Aged 18 years or older.
2. Diagnosis of MS according to revised McDonald criteria (35).
3. Written informed consent to participate in the study.
4. An Expanded Disability Status Scale (EDSS) (36) score between 2.5 and 7 and a score of ≥2 points in two of the following functional systems (FS): pyramidal, brainstem or cerebellum.
5. ≥1 point in the cognitive FS of the EDSS.
6. Ability to engage and manage new technologies and use the headset.
7. Own a compatible smartphone to install the VR program.
8. Patients requiring medication which, in the investigator's opinion, may interfere with rehabilitation results, must be on a stable regimen at study entry. Note: Patients in whom this medication is modified during the study will be excluded from the study.

Patients who could benefit from a rehabilitation program.

Subjects who meet any of the following criteria will be excluded from study entry:

1. Participation in any clinical trial at the time of data collection.
2. Cognitive impairments which, in the investigator's opinion, might pose difficulties for understanding and completing the study questionnaires or operating the VR system.
3. Have had a relapse and/or have received methylprednisolone or equivalent within the last 30 days or during the study. Note: Patients who have a relapse or who are treated with this medication during the study, as described above, will be discontinued from the study.
4. Have initiated pharmacological treatments that could modify the patient's walking ability within the last 30 days or during the study. Note: Patients who are treated with medication during the study, as described above, will be discontinued from the study.
5. Any visual or hearing disorder that prevents correct use of the VR system.
6. Diagnosis of any health condition which, in the investigator's opinion, prevents the completion or continuation of the rehabilitation program.

### Intervention

Patients will attend 8 on-site rehabilitation sessions during 4 weeks (2 sessions per week) of ~2 h duration each. The rehabilitation sessions will be conducted by a physiotherapist in groups of a maximum of 4 at the physiotherapy room of the Hospital Universitario de la Princesa in Madrid (Spain). Groups will be formed according to patients' functional level measured by the EDSS (36), resulting in three subgroups (low, medium, or high functional level) in each rehabilitation group (VRR and CR). The number of sessions conducted on site will provide sufficient training to allow patients to continue performing the rehabilitation exercises at home independently.

### Conventional Rehabilitation

#### Physical Exercises

All patients will receive CR on site (eight sessions in the CR group and four sessions in the VRR group). The rationale for including four sessions of CR in the VRR group is to ensure that all patients become familiar with all the exercises and learn to perform the movements correctly before doing them within the virtual environment. Patient status and suitability to perform the exercises (heart rate, blood pressure, temperature, and fatigue) as well as the temperature and humidity of the room will be evaluated at the beginning of each session.

During the rehabilitation sessions, exercises will be based on a standardized rehabilitation program (37) which has been extensively described elsewhere. Briefly, to ensure appropriate muscle activation, a 30-min warm-up with light aerobic activity and mobilization using the cycle ergometer or pedal exerciser will be performed. This will be followed by stretching, and exercises aimed at improving spasticity (~10–15 min), coordination and balance (~10 min), mobilization (~10–15 min), breath control (~10–15 min), and gait (~10–15 min). The exercises will be conducted in supine, prone, quadruped, knees and standing positions, and they will be interspersed with rest periods. Energy-saving techniques will be also taught during the sessions.

All exercises will be individually tailored according to the patient's abilities and needs. Exercises are classified according to the level of difficulty (low, medium, high), and patients will be assigned to a group (low, medium, and high function) based on an initial evaluation. Given that all patients in the group will have a similar level of function, patients will easily adapt to the pace of the session. During all sessions, patients will be led and supervised by the physiotherapist, who will give personalized instructions on the exercises according to the patient's function and ensure all the exercises are performed safely. For the last session, patients will be engaged to attend accompanied by a family member in order to serve as a reinforcement for rehabilitation at home. Patients will be requested to perform the exercises at home in the presence of a family member and after having removed all possible obstacles in the play area to increase patient safety.

#### Cognitive Exercises

The VRR program includes in its design a component of cognitive rehabilitation. Tasks performed in the virtual environment are designed to train alternating and sustained attention based on the Sohlberg and Mateer model (38). Patients in the CR group will perform a cognitive task that will allow them to stay cognitively active. They will be provided with a notebook containing several texts selected by a neuropsychologist and tailored to patient deficits. Patients will be required to read and summarize the text in ~ two lines, and they will be instructed to do one text each day they perform the physical exercises at home. This task is not based on any neuropsychological model of attention training.

#### Virtual Reality Rehabilitation

The software and hardware that will be used in the VRR group are discussed below. Briefly, a program with task-specific interactive games was specifically designed for MS patients by the multidisciplinary team involved in rehabilitation. Its design was based on the type of exercises performed during CR that is routinely conducted at the hospital and that has previously shown to be effective in improving motor symptoms in MS patients. The software allows the physiotherapist to select the exercises and level of difficulty of the exercises to be performed by the patient, and to follow the patient's progress. The hardware is composed of devices for visualization and recognition of body movements that are considered easy to use in the rehabilitation context.

#### Hardware

The VR system that will be used for visualization on-site is the HTC Vive headset (HTC Corporation, China). This VR headset uses stereoscopy, a technique to create the illusion of depth from the analysis of two images obtained through binocular vision. The lighthouse tracking system (Valve corporation, USA) and the HTC Vive Tracker will be also used to recognize body movements. Four Vive Trackers can be attached to the patient's wrist and ankles (one at each end) to enable body tracking while leaving the patient's hands free, which will be required for some exercises. This system allows for including virtual feet in the virtual environment that represent the movements of the patient's feet in real time.

The HTC Vive has some limitations to be used at home, such as specific hardware requirements. Therefore, the Oculus Quest headset will be used instead, which is a fully standalone headset that includes two ergonomic controllers. The virtual environments have been adapted for the Oculus Quest headset. Since the Oculus Quest headset cannot detect the patient's feet, exercises in which feet had to be identified were modified. Both the HTC Vive and Oculus Quest headsets increase the patient's sense of immersion into virtual environments and allow the system to execute a cause-effect response between exercise storytelling and the patient's response. Both headsets incorporate a system that warn the patient when she/he approaches the physical limits of the rehabilitation area, so that the patient is aware of them.

#### Software

**Figure 2:**
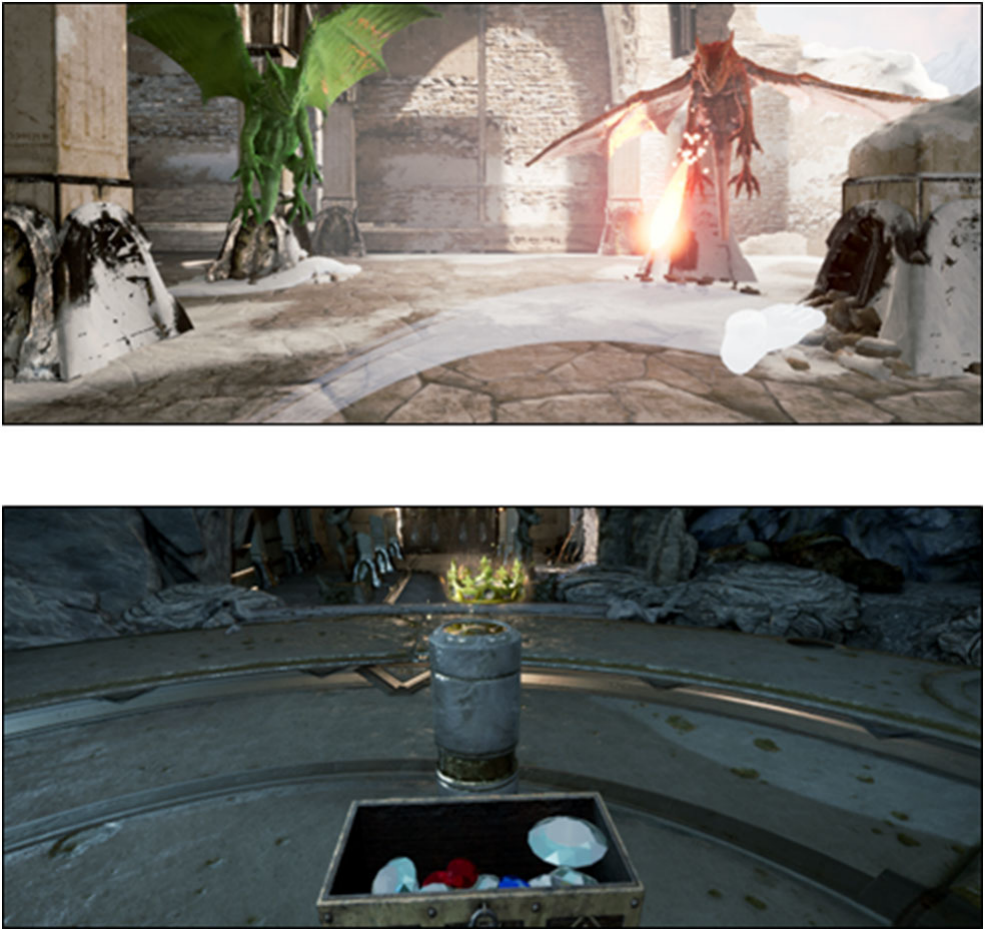
Screen shots of the *fantasy medieval world* virtual environment.

**Figure 3:**
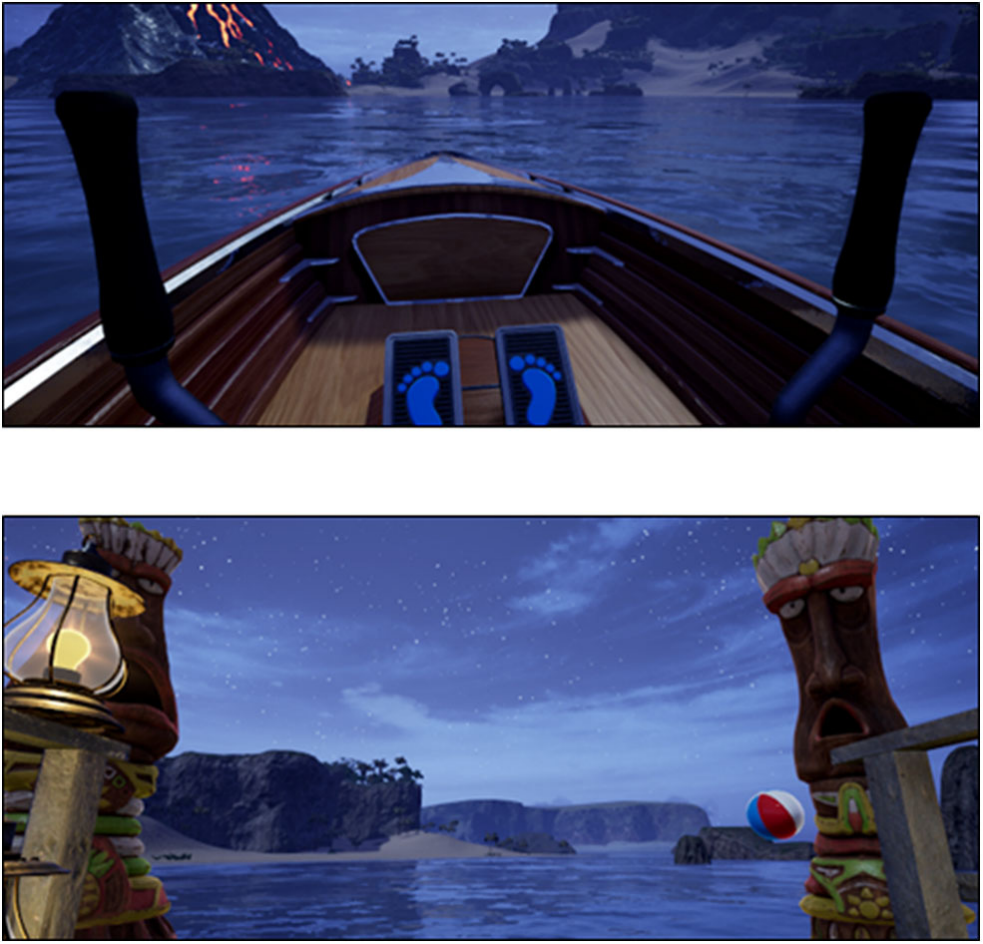
Screen shots of the *deserted island* virtual environment.

The program consists of two virtual environments that are designed and developed to allow the patient to perform motor and cognitive rehabilitation exercises in an interactive game-based setting. The patient will be required to perform specific tasks in order to accomplish a mission in two different environments: a *fantasy medieval world* (Figure 2) and a *deserted island* (Figure 3). These virtual environments were designed to combine a variety of rehabilitation exercises in different positions (sitting, standing or lying) with gaming elements, making the otherwise monotonous exercises more competitive and motivating.

The patient will practice various movements (kick forward, kick back, triple flex, paddle) while performing the required tasks. Some examples of the tasks in the virtual environments are fighting against flying dragons that throw fireballs that patients have to return by kicking them, learning how to use a sword in a fencing class, or rowing in a small boat to escape from pirates who attack the island. The movements incorporated in these tasks were suggested by the multidisciplinary team and were mainly intended to decrease fatigue, and improve spasticity control, balance and coordination. The cognitive components of the tasks are aimed at training several cognitive domains, such as attention and short-term memory. For instance, one of the tasks is an alchemy class in which the patient is required to cook a recipe with the exact same ingredients in a cauldron. Before each task starts, an avatar called Guía will explain to the patient in detail the steps required to complete the task, while another avatar called Cidoimos will do a physical demonstration on how to perform the task, so that the patient can copy the specific position and movements made by Cidoimos in order to accomplish the goal explained by Guía (see Supplementary Material 1). These avatars will serve as a support tool for patients to conduct the exercises that will be previously explained and supervised by the physiotherapist.

The environments were implemented using the Unreal Engine 4 (Epic Games, USA) game engine, which is compatible with both HTC Vive and Oculus Quest headsets. A main user interface was developed for the HTC Vive to allow the physiotherapist to: (i) include the list of patients and record the date of their last session, (ii) customize a list of exercises for each session according to the patient level and progress, (iii) consult the exercises completed and not completed, (iv) keep a record of total time spent in the session and specific time spent on each exercise.

During each on-site VRR session, the physiotherapists will supervise whether patients correctly perform all the exercises. The exercises have been classified in three categories according to their physical and cognitive difficulty (A, B, or C, corresponding to a low, medium and high level of difficulty). The level of difficulty and the type and number of physical exercises will be selected based on the functional status of each patient. The difficulty of the cognitive tasks will be automatically adjusted according to patient progress. Thus, a patient could simultaneously perform physical exercises of medium difficulty and cognitive tasks of high difficulty. The accuracy with which the patient performs the task will constitute the measure of game performance. Positive feedback will be provided by the system before, during, and after task performance to boost patient motivation. Patients will be encouraged to contact any member of the study team in case of discomfort, for any question regarding the study protocol, or in case of technical problems.

### Outcome Measures

Table 1 lists the study data collection overview, including primary and secondary outcome measures. Study variables will be collected on an electronic case report form. The assessments are described below.

**Table 1:** Data collection overview.

**Data collection**	**Screening visit (V_**0**_)**	**Baseline visit (V_**1**_)**	**Post-rehabilitation on-site visit (V_**2**_)**	**Post-rehabilitation at-home or study completion visit (V_**3**_)**
Informed consent	x			
Inclusion and exclusion criteria	x			
Sociodemographic data: age, sex, educational level and profession	x			
Clinical history: time from MS diagnosis, type of MS, use of DMTs, previous EDSS score, clinical rehabilitation history	x			
Satisfaction with VRR: USEQ	x	x	x	x
Adherence to rehabilitation		x	x	x
ClinROs: EDSS, 9-HPT, T25-FW, 6 MWT, MAS, BBS		x	x	x
PROs: MSIS-29, EuroQoL-5D, MFIS, Barthel index, MSWDQ-23, BDI-FS, HADS, PSFS, MSNSQ		x	x	x
Rao's BRB		x	x	x
Discomfort		x	x	x

#### Primary Outcome Measures

The primary outcome is satisfaction at 6 months of VRR program initiation. Satisfaction is assessed by using the USEQ, which is a 6-item questionnaires using a 5-point Likert scale (29). The primary variable is the USEQ total score, which ranges from 6 (lower satisfaction) to 30 points (higher satisfaction). To calculate this total score, all of the questions are considered to be positive, except for Q5, which is considered to be negative. The numerical value of the positive questions is used to calculate the score. The negative question subtracts the numerical value of the response from 6 and then adds this result to the total score (for instance, if the patient selects two in Q5, then four is added to the total score). The questions and their scores are shown in Table 2.

**Table 2:** The User Satisfaction Evaluation Questionnaire (USEQ).

**Question**	**Response (not at all-very much)**
1. Did you enjoy your experience with the system?	1 2 3 4 5
2. Were you successful using the system?	1 2 3 4 5
3. Were you able to control the system?	1 2 3 4 5
4. Is the information provided by the system clear?	1 2 3 4 5
5. Did you feel discomfort during your experience with the system?	1 2 3 4 5
6. Do you think that this system will be helpful for your rehabilitation?	1 2 3 4 5

The questions that composed the USEQ come from the set of questions of the Suitability Evaluation Questionnaire (SEQ) that evaluate satisfaction (39). The SEQ is a previously developed 14-item questionnaire designed to test satisfaction, acceptance, and security of use in VRR systems. The SEQ has previously been used in a clinical trial of MS patients receiving VRR conducted in Spain (24).

Probably due to the relatively recent publication of the USEQ (July 2017), no study using this questionnaire in MS patients has yet been published. We selected the USEQ for four main reasons. Firstly, because it has been specifically designed to assess satisfaction with a VRR program. Secondly, because it has been validated in a Spanish population of patients with balance disorders. Thirdly, because the patients considered the questionnaire to be short and easy-to-understand. And lastly, because the questionnaire demonstrated to be reliable and to have adequate internal consistency (α = 0.716) (29).

### Secondary Outcome Measures

#### Rehabilitation Adherence

Adherence to the on-site rehabilitation sessions will be measured by the number of rehabilitation sessions attended. Participants will be contacted by email or phone if they fail to take part in two consecutive rehabilitation sessions. Rehabilitation adherence at home will be measured by the VR system itself in the VRR group, and by the nurses participating in the study who will require patients in the CR group to indicate via email the frequency with which they have conducted the exercises each week.

#### Clinician-Reported Outcomes

A variety of clinician-reported outcomes (ClinROs) will be collected at the baseline visit (V_1_), post-rehabilitation on-site visit (V_2_), and a post-rehabilitation at-home visit or study competition visit (V_3_) by a neurologist unaware of the rehabilitation group allocation. Changes from the baseline visit (V_1_) to month 6 (V_3_) and from the baseline visit (V_1_) to month 1 (V_2_) will be collected as secondary and exploratory variables, respectively. ClinROs were selected on the basis of previous studies of rehabilitation that reported statistically significant improvements by using VRR in disability (17, 18), spasms and spasticity (40), and balance (19, 24, 33).

Disability progression will be evaluated by the EDSS (36), the Nine-Hole Peg Test (9-HPT) (41), the Timed 25-Foot Walk (T25-FW) (42), and the Six-Minute Walk Test (6 MWT) (43). The EDSS (36) assesses 7 functional systems based on a neurological examination. The score ranges from 0 to 10 in 0.5-unit increments that represent higher levels of disability. The 9-HPT (41) tests upper extremity function. Patients will be instructed to place pegs from a container one by one into each of the nine holes of the board as quickly as possible. The score is the average time required in four trials (two trials for each hand). It is one of the three components of the MS Functional Composite (MSFC) disability assessment (42). No specific cut-off score has been validated for MS (44), but a worsening of 20% on all the measures of the MSFC is generally accepted to indicate disease progression. The T25-FW (42) evaluates mobility and leg function performance based on the time until the patient reaches the 25-foot mark (7.62 m). Patients will start at a line on the floor and will be instructed to walk as quickly as possible but safely beyond the second line 25 feet away. The score is the averaged time of 2 trials, and the time limit per trial is 3 min. It is another of the three components of the MSFC disability assessment (42). The 6 MWT (43) measures the distance walked over a span of 6 min. Patients will be instructed to walk at a comfortable pace back and forth along a 60-foot walkway for 6 min (43).

Spasms and spasticity will be assessed by the Modified Ashworth Scale (MAS) (45). This scale measures resistance during passive soft-tissue stretching. It consists of a 5-point nominal scale using subjective clinical assessments of tone ranging from 0 to 4.

Balance will be evaluated by the Berg Balance Scale (BBS) (46). The BBS assesses 14 daily life activities with scores ranging from 0 to 4. The cumulative results categorize patients into three groups: ≤20 for wheelchair users, >20 ≤ 40 for those walking with assistance, and >40 ≤ 56 for those who are independent.

#### Patient-Reported Outcomes

Several patient-reported outcomes (PRO) will be also collected at the baseline visit (V_1_), post-rehabilitation on-site visit (V_2_), and post-rehabilitation at-home visit or study competition visit (V_3_). Changes from the baseline visit (V_1_) to month 6 (V_3_) and from the baseline visit (V_1_) to month 1(V_2_) will be collected as secondary and exploratory variables, respectively. PROs were selected on the basis of previous studies of rehabilitation that reported statistically significant improvements by using VRR in HRQoL (22, 26, 47, 48), fatigue (19, 49), and ADL (26). The evaluation of depression and anxiety after VRR has received little attention, despite the fact that the prevalence of these disorders has increased (50). To evaluate depression and anxiety, we selected questionnaires that have shown to be valid measures of these disorders in MS (51, 52). As far as we know, no previous study has evaluated work status and self-perceived changes after a VRR program. Therefore, questionnaires specifically designed for MS patients in this regard (46, 47) were considered the most appropriate to be used here (53, 54). The Spanish for Spain version of all PRO will be used, and all PRO assessments will be collected by an electronic device.

HRQoL will be assessed by the Multiple Sclerosis Impact Scale (MSIS-29) (55) and the EuroQol EQ-5D-5L (56). The MSIS-29 (55) measures the physical and psychological impact of MS from the patient's perspective. It is composed of 29 items grouped in two scales: physical and psychological. Each item is assessed by the patient using a 5-point Likert scale ranging from 1 (not at all) to 5 (extremely). Each of the two scales are scored by summing the responses across items, then converting them to a 0–100 scale where 100 indicates greater impact of disease on daily function (worse health). The MSIS-29 has shown to be one of the most sensitive scales in detecting rehabilitation-induced changes (48). The EQ-5D-5L (56) consists of a five-item descriptive system and a visual analog scale (EQ VAS). The descriptive system consists of five health dimensions (mobility, self-care, usual activities, pain/discomfort and anxiety/depression) and subjects may choose from five response levels (no problems =1, slight problems = 2, moderate problems = 3, severe problems = 4, and unable to/extreme problems = 5), where higher values indicate worse health.

Fatigue will be measured by the Modified Fatigue Impact Scale (MFIS) (57). The MFIS is based on 21-items derived from interviews with MS patients concerning how fatigue impacts their lives. This instrument provides an assessment of the effects of fatigue in terms of physical, cognitive, and psychosocial functioning. The total score of the MFIS ranges from 0 to 84 (physical, 0 to 36; cognitive, 0 to 40; and psychosocial, 0 to 8).

ADLs will be evaluated by the Barthel index (58). The Barthel index is a 10-item ordinal scale that uses ten variables describing ADLs and mobility. Each item is rated on this scale with a given number of points assigned to each level or ranking. A higher number is associated with a greater likelihood of being able to live at home with a degree of independence.

Work status will be assessed by the Multiple Sclerosis Work Difficulties Questionnaire (MSWDQ-23) (53, 59). The MSWDQ-23 is a brief valid measure of workplace difficulties that can predict both employment outcomes and expectations in patients with MS. It examines work difficulties across three broad domains: psychological/cognitive barriers (11 items), physical barriers (eight items), and external barriers (four items). Participants are asked to rate how often they experienced each difficulty as a result of their MS over the past 4 weeks on a 5-point Likert-type scale, with response options ranging from 0 = “Never” to 4 = “Almost Always.”

Depression will be tested by the Beck Depression Inventory-Fast Screen (BDI-FS) (60). The BDI-FS is a 7-item questionnaire designed to evaluate depression in patients with medical illness (dysphoria, anhedonia, suicidal ideation, and cognition-related symptoms) on a 3-point scale. Scores on the BDI-FS range from 0 to 21, with higher scores indicating more depressive symptoms.

Anxiety will be measured by the Hospital Anxiety and Depression Scale (HADS) (61). The HADS is a 14-item questionnaire designed to evaluate anxiety and depression in patients with medical illness on a 3-point scale. It provides two sub-scales, one for anxiety and one for depression symptoms. Scores for every subscale range from 0 to 21, with higher scores indicating more anxiety and depression symptoms.

Spasm frequency will be evaluated by the Penn Spasm Frequency Scale (PSFS) (62). The PSFS is a two component self-report measure of the frequency of reported muscle spasms commonly used to quantify spasticity. The first component is a 5-point scale assessing the frequency with which spasms occur ranging from 0 to 4. The second component is a 3-point scale assessing the severity of spasms ranging from 1 to 3.

The Multiple Sclerosis Neuropsychological Screening Questionnaire (MSNSQ) (54) will be used for identifying patients at high risk for cognitive impairment in MS.

#### Cognitive Assessment

To test the cognitive performance of the participants, Rao's Brief Repeatable Battery (BRB) will be applied by a neurologist unaware of the rehabilitation group allocation. This battery has shown to be a sensitive measure of cognitive impairment in MS patients (63). It consists of the Selective Reminding Test (SRT), the 10/36 Spatial Recall Test (SPART), the Symbol Digit Modalities Test (SDMT), the Paced Auditory Serial Addition Test (PASAT) and the Word List Generation Test (WGT).

#### Discomfort

Each patient will be asked about the presence of discomfort (e.g., dizziness, vertigo, nausea, headaches, falls, and others) by the physiotherapist after each session. During the at-home rehabilitation period, patients will be instructed to record the occurrence of discomfort, and to report it to the treating neurologist at each study visit. Discomfort will also be assessed by question 5 in the USEQ (see Table 2).

#### Demographic and Clinical Characteristics

Sociodemographic data (age, sex, educational level and profession) and clinical data (time from MS diagnosis, type of MS, use of DMTs, previous EDSS score, and clinical rehabilitation history) will be also collected.

### Data Analysis

Data entered manually will be collected via electronic data capture (EDC) by the site. PRO data will be collected by patients on electronic questionnaires implemented in an electronic device. The data from the questionnaires will be automatically transferred to the EDC system.

### Sample Size Calculations

Sample size calculations were conducted using the Granmo v7.11 software. No previous studies using the USEQ in patients with MS have been performed, so assumptions had to be made for estimation of the sample size. Since the standard deviation (SD) of the USEQ score in MS patients was not available, an estimation based on the maximum variance from the range SD=range/2 was considered. In the particular case of the total score of the USEQ, an estimated SD of 12 (in the most unfavorable case) was used. Considering a SD of 12 units, 32 patients in the VRR group will be sufficient to estimate a population mean of the total score of the USEQ with 95% confidence and a precision of ±4.3 units. A replacement rate of 5% has been anticipated.

Comparisons between the rehabilitation groups (VRR vs. CR) are planned for some secondary and exploratory outcomes. These objectives are assessed by scales or questionnaires that can be transformed to a 0–100 scale. In a scale of this range, a SD of 50 points for the mean of both groups can be assumed by the maximum variance principle. A difference of 20 points in the mean change between the two groups and a correlation coefficient of 0.90 will be assumed for each scale/questionnaire. Accepting an alpha risk of 0.05 and a beta risk of 0.2 in a two-sided test, 16 patients in the CR group and 32 patients in the VRR group are required to recognize a difference equal to or >20 units as statistically significant. A drop-out rate of 5% has been anticipated.

### Statistical Analysis Plan

To address the primary objective, the total score for USEQ in the VRR group with measures of central tendency and dispersion (mean, SD, median, minimum and maximum) will be presented.

To compare mean changes after 6 months of VRR or CR on domains typically affected by MS (HRQoL, fatigue, ADL, work status, depression, anxiety, disability, spasms and spasticity, balance, and cognition) an inferential analysis will be used. For variables that follow a normal (or parametric) distribution, a *t*-test will be used. For variables that do not follow a normal (or parametric) distribution, Mann-Whitney (for unpaired data) or Wilcoxon (for paired data) hypothesis tests will be used. For the analysis of the contingency tables and comparison of proportions and/or frequency distributions, the chi-squared test (or Fisher's exact test where appropriate) will be used. All tests will be 2-tailed, and *p* < 0.05 will be considered significant.

To investigate therapy adherence and discomfort during the VRR program, a descriptive statistical analysis will be performed. Measures of central tendency and dispersion (mean, SD, median, minimum and maximum) will be presented.

The association between clinical and demographic characteristics and patient satisfaction with the VRR program will be calculated using Pearson or Spearman correlation coefficients, as appropriate.

Absent data will not be accounted for and will be considered missing data. No imputation will be done. Parameter estimates and 95% confidence intervals will be reported for the principal quantitative outcomes. The Statistical Package for the Social Sciences (SPSS) software (v 22 or later) will be used to conduct the analysis. Further details of data analysis will be provided in a statistical analysis plan.

## Discussion

Despite the great advances in the number and effectiveness of DMTs to reduce relapse rates and slow disease progression (64), disability often continues to worsen and adjunct non-pharmacological treatments aimed at managing the symptom burden seem imperative. Fatigue and changes in motion, cognition and mood are commonly reported by MS patients, and these symptoms negatively affect patients HRQoL by interfering with their ability to work and pursue leisure activities (65). Rehabilitation programs facilitate the learning of new strategies that allow patients to improve abilities in activities of daily living and maintain a higher level of independence and self-empowerment.

An integrated approach with cognitive and motor training has demonstrated to be associated with improvements not only in cognitive and motor performance but also in mood (66). Constant repetition of the same movements, as occurs in CR, might result in the patient being less engaged and motivated, which will affect therapy adherence and ultimately reduce rehabilitation effectiveness. The emergence of an increasing number of VRR programs enables simultaneous training of physical, cognitive, and psychological aspects in an immersive environment that shows great potential to improve patient motivation and adherence (67, 68), mainly due to the gamification and personalized feedback. However, in order to systematically adopt VRR programs in routine clinical practice, information on user satisfaction and not only effectiveness and efficiency of the program is required. This pilot study aims to evaluate MS patients' satisfaction with a novel VRR program using the USEQ, a questionnaire that was specifically designed to evaluate satisfaction in VRR systems (29).

A recent systematic review has concluded that the benefits of VR rehabilitation reported in the literature are usually observed in a context where VR devices were customized for people with neurological disabilities (68). Our VRR program is the result of the adaptation by a multidisciplinary group of experts of the CR protocol of the Rehabilitation Department, which was developed jointly by neurologists, physiatrists, neuropsychologists and physiotherapists at the Hospital Universitario de la Princesa to decrease fatigue and improve function in patients with different degrees of disability due to MS. The software has been specifically designed for these patients by experts in the design and technological development of gamified and narrative systems. This adaptation is an attempt to create an immersive physical rehabilitation program while simultaneously incorporating cognitive tasks to improve cognitive impairments that are usually prevalent in these patients but that are not commonly targeted in CR.

### Strengths and Limitations

The VRR program proposed in this pilot study has several strengths. Firstly, it has a multidisciplinary approach, which could be expected to have a profound impact on optimization of symptoms management in MS patients (69). Secondly, the VRR is designed to be used remotely, which will increase outreach to those who might otherwise have difficulties to access rehabilitation, such as those with limited geographic accessibility, those unable to reconcile working hours and therapy schedule, or those dependent on others to arrive at the treatment center. VRR is not intended to replace CR but to complement it. Modifications to the rehabilitation modality can be made based on the specific patient situation. Thirdly, although VRR requires an initial economic investment, it could reduce long-term healthcare costs compared to traditional face-to-face rehabilitation (70). Fourthly, because the VR tasks were specifically designed for patients with MS, injuries observed in rehabilitation programs that used exergames (22) are not expected to occur. And lastly, patients will be followed up for 6 months, which represents a considerably longer period than that included in previous studies (18, 19, 26, 33, 34). Furthermore, the inclusion of a group of patients receiving CR will allow us to evaluate comparative changes between groups on a wide range of domains typically affected by MS. The information provided by the current study will be highly relevant for considering the systematic implementation of this VRR program in routine clinical practice of Spanish hospitals or clinical rehabilitation centers.

Limitations of this study are related to the small sample size of MS patients from a single center and the limited follow-up. However, sample size calculations determined that the sample size will be sufficient to evaluate the study objectives, and the follow-up period is even longer than that included in previous similar studies. Another limitation of the study could be the differences between the VRR system used on site and the system used at home, the latter being less complete than the former, although it includes all the necessary tools to conduct the required tasks. This pilot study will be suitable for generating further hypotheses related to VRR in MS.

## Dissemination of Research Results

Following completion of the study, a manuscript will be prepared to report the primary, secondary and exploratory outcomes. The manuscript will be reviewed and approved by all the authors prior to submission. Each study participant will receive a summary of the study results under request.

## Study Status

The study is ongoing and has not completed participant recruitment at the time of submission (the study has enrolled 50% of study participants).

## Ethics Statement

The studies involving human participants were reviewed and approved by Comité Ético Hospital La Princesa. The patients/participants provided their written informed consent to participate in this study.

## Author Contributions

VM-L, WA, FG-V, DG, TH, AP, NT, and AV-D participated in the design and/or optimization of the VRR protocol. All authors read, approved the final manuscript, contributed to conception of the study design, drafting the manuscript, and critically revising it for intellectual content.

## Conflict of Interest

This study has received funding from Roche Pharma S.A. CG-B, JM, NM, and DP are employees in the Medical Department of Roche Pharma. They do not hold any stocks or shares in Roche Pharma that may, in any way, gain or lose financially from the publication of this manuscript. The remaining authors declare that the research was conducted in the absence of any commercial or financial relationships that could be construed as a potential conflict of interest.

## Supplementary Material

Supplementary Material 1 Virtual reality environments. The software was created based on the exercises from a conventional rehabilitation program by an expert team in creating virtual reality experiences, including technological design, narrative design, gamification system design, and audiovisual design. The software has been designed for the patient to experience the rehabilitation session as a narrative in which he/she is the protagonist of an adventure that will take place in two different environments (a fantasy medieval world and a deserted island) where the patient is presented with several missions. To achieve the goal of each mission, the patient must perform the mechanics of the physical or cognitive exercises designed by the medical team. The patient, accompanied by two characters (Guía and Cidoimos) will be given information about the mission, the environment and how to perform the challenge of the exercise during the trip that represents each therapeutic session. The implementation of these characters, which interact with the patient within a narrative framework where he/she is the protagonist of the story, have been designed taking into account the MS patient's needs. A button system has also been designed that allows the user to determine key aspects of the session, such as listening again to the guide's instructions (if the patient has not understood the mission), or the possibility of skipping these instructions (if the patient already knows the challenge in question). Having the two characters (Guía and Cidoimos) who accompany the patient in each adventure and the fact that the environment detects the patient's presence are two relevant factors that enable the creation of an immersion experience.
